# Anticipating the potential for positive uptake and adaptation in the implementation of a publicly funded online STBBI testing service: a qualitative analysis

**DOI:** 10.1186/s12913-018-2871-x

**Published:** 2018-01-30

**Authors:** Cathy Chabot, Mark Gilbert, Devon Haag, Gina Ogilvie, Penelope Hawe, Vicky Bungay, Jean A. Shoveller

**Affiliations:** 1 School of Population and Public Health, University of British Columbia, 2206 East Mall, Vancouver, BC V6T 1Z3 Canada; 2 Clinical Prevention Services, British Columbia Centre for Disease Control, 655 West 12th Avenue, Vancouver, BC V5Z 4R4 Canada; 3 Menzies Centre for Health Policy and The Australian Prevention Partnership Centre, Charles Perkins Centre (D17), The University of Sydney, Sydney, NSW 2006 Australia; 4 O’Brien Institute for Public Health, Cumming School of Medicine, University of Calgary, 3280 Hospital Drive NW, Calgary, AB T2N 4Z6 Canada; 5 School of Nursing, University of British Columbia, 111-2176 Health Sciences, Vancouver, BC V6T 1Z3 Canada

**Keywords:** Internet-based intervention, Online STBBI testing, Qualitative research, Implementation context, Agency

## Abstract

**Background:**

Online health services are a rapidly growing aspect of public health provision, including testing for sexually transmitted and other blood-borne infections (STBBI). Generally, healthcare providers, policymakers, and clients imbue online approaches with great positive potential (e.g., encouraging clients’ agency; providing cost-effective services to more clients). However, the promise of online health services may vary across contexts and be perceived in negative or ambiguous ways (e.g., risks to ‘gold standard’ care provision; loss of provider control over an intervention; uncertainty related to budget implications). This study examines attitudes and perceptions regarding the development of a novel online STBBI testing service in Vancouver, Canada. We examine the perceptions about the intervention’s potential by interviewing practitioners and planners who were engaged in the development and initial implementation of this testing service.

**Methods:**

We conducted in-depth interviews with 37 healthcare providers, administrators, policymakers, and community-based service providers engaged in the design and launch of the new online STBBI testing service. We also conducted observations during planning and implementation meetings for the new service. Thematic analysis techniques were employed to identify codes and broader discursive themes across the interview transcripts and observation notes.

**Results:**

Some study participants expressed concern that the potential popularity of the new testing service might increase demand on existing sexual health services or become fiscally unsustainable. However, most participants regarded the new service as having the potential to improve STBBI testing in several ways, including reducing waiting times, enhancing privacy and confidentiality, appealing to more tech-savvy sub-populations, optimizing the redistribution of demands on face-to-face service provision, and providing patient-centred technology to empower clients to seek testing.

**Conclusions:**

Participants perceived this online STBBI testing service to have the potential to improve sexual health care provision. But, they also anticipated actions-and-reactions, revealing a need to monitor ongoing implementation dynamics. They also identified the larger, potentially system-transforming dimension of the new technology, which enables new system drivers (consumers) and reduces the amount of control health care providers have over online STBBI testing compared to conventional in-person testing.

## Background

Internet-based population health interventions are a new and growing area of public health service provision; yet, to date, there has been limited research regarding the implementation context of internet-based health services. Published accounts have focused primarily on the adoption of electronic medical records [1–3]. This study takes place in Vancouver, British Columbia (BC), Canada, where diagnosis rates of sexually transmitted infections (STI) are high and rising among some populations, including young people under 25 and men who have sex with men (MSM). In BC, the rate of genital chlamydia increased to 288.4 per 100,000 population in 2014, continuing the overall provincial trend of a steady increase since 1998. Similarly, diagnosis rates of gonorrhea have increased among youth under 25 in the past decade [4]. Diagnosis rates of other STI are high among gay, bisexual, and other MSM in BC. For instance, in 2014, 84.9% of all new syphilis cases (466 of 549 new cases) occurred in MSM [4]. MSM also accounted for the greatest number of new positive Human Immunodeficiency Virus (HIV) diagnoses (58%) in BC in 2014 [5].

Despite these high rates of new infections, testing services for sexually transmitted and other blood-borne infections (STBBI) remain under-accessed by many groups, both in Canada and internationally [6–8]. As a result, public health services are developing new ways to promote testing uptake, with the hope that it will enhance timely treatment, lower rates of onward transmission, and reduce adverse health outcomes [9, 10]. Access to internet-based testing is provided in a variety of ways, ranging from publicly funded mail-in testing kits for chlamydia and HIV in the United Kingdom [11], to commercial, fee-for-service STBBI testing in some regions of Australia [12], the United States [13–15], and Canada [16, 17]. And, healthcare providers (HCP), policymakers, and clients frequently regard online health services as having the potential to reduce access barriers and service delivery costs, while contributing to patient self-care regimens [18–22].

### GetCheckedOnline

GetCheckedOnline (GCO) is a new internet-based STBBI testing service developed by the British Columbia Centre for Disease Control (BCCDC) in Vancouver, Canada [23], and the first of its kind in Canada to offer comprehensive online testing for STBBI. GCO is a virtual extension of the provincial STI clinic and is integrated with existing clinical and public health services. As such, the aim of GCO is to *complement* – not replace – existing face-to-face clinical services, with the goal of increasing testing uptake and facilitating earlier diagnosis and treatment, particularly among MSM and young people [17]. By providing an internet-based option to asymptomatic persons to test, GCO also aims to result in “more focused use of clinical nursing and physician resources for clients requiring clinical diagnosis and treatment, and increased capacity for drop-in visits” at BCCDC STI clinics [24]. Its development is aligned with the BC Ministry of Health, which advocates increasing implementation of patient-centred care services, such as self-management of healthcare, patient and HCP shared decision-making, increasing patients’ access to information, and advancing health promoting behaviours [25].

GCO is provided free of charge to all users and is currently available in Vancouver and select communities in two other BC health regions. Users create a secure online account before initiating an assessment of their risk of exposure to STBBI. Users complete an online informed consent process to indicate that they understand what STI they are being tested for, how they will receive their test results, and how to access more information [26]. Users are then instructed to print a laboratory requisition that includes orders for chlamydia, gonorrhea, syphilis, HIV, and (for some clients) Hepatitis C tests. Clients take their printed requisition to a participating laboratory, where blood and urine samples (and in some cases, self-collected oral and rectal swabs) are collected and then transferred to the provincial Public Health Lab for analysis. All users test under an alphanumeric code in place of first and last names. Specimen analysis is typically completed within seven to 12 days, whereupon clients are prompted via email to sign into their GCO account to view their test results. Clients are able to view their negative test results online. If they test positive or have an inconclusive result, the website prompts them to contact the provincial STI clinic at BCCDC by phone; at the same time, BCCDC nurses will attempt to contact any client with a positive result. At this point, all clients with inconclusive or positive lab results are treated as all other BCCDC clients with a similar result, where results are discussed by phone and the individual is referred to a BCCDC clinic for further testing or treatment, as warranted [14]. Clients living in select communities in the two other health regions where GCO is available are referred to public health clinics, walk-in clinics, or pharmacies that have partnered with the health authority to provide treatment to GCO clients. Overall, GCO reduces the interaction with the traditional health care system by removing the need for an in-person pre-test visit at a clinic to obtain a requisition for testing, and a post-test visit for the majority of clients who have a negative result. Clients are still required to present to a laboratory for specimen collection.

GCO was first piloted in September 2014 and modifications were made based on feedback from users and BCCDC staff, as well as early evaluation of the intervention. The risk assessment questions were revised in Autumn 2015 to be more appropriate for clients of all gender identities and to allow for the prospective collection of variables needed to validate clinical prediction rules for gonorrhea, chlamydia, and HIV testing. In January 2016, self-collected throat and rectal swab testing for chlamydia and gonorrhea was added based on feedback from clinician consultations and to reflect clinical practice guidelines [27]. GCO developers continue to adapt the intervention, based on ongoing monitoring and evaluation across contexts. BCCDC’s Online Sexual Health Services (OSHS) coordinator has overseen the development and delivery of GCO across health authority regions, which implicates contextual features of geography (e.g., rural; urban), epidemiology (e.g., STI ‘hot spots’ and areas with below average STI rates), and varying perspectives on the potential value of GCO. The provincial Ministry of Health and regional health authorities are generally supportive of GCO and have identified local champions [25] who help shape GCO’s adaptation and gradual expansion.

### The implementation context of GCO

In order to understand the implementation context into which GCO was being introduced, one must understand how STBBI testing and treatment services are provided in BC. Context is “a constellation of active interacting variables” that are unique to the implementation of a particular intervention [28]. In British Columbia, STBBI testing and treatment services are a complex system comprised of individual agents (e.g., healthcare providers, administrators and policymakers, support staff, and clients) that have the freedom to act in ways that are not always predictable, and whose actions are interconnected so that one agent’s actions may alter the context for other agents [29]. For instance, it is important to consider factors such as innovations in STBBI testing technologies (e.g., urine-based NAAT testing, rather than urethral swabs, are the current standard of care) and treatment policies (e.g., routine implementation of partner delivered therapy), as well as changes in settings (e.g., staffing changes at clinics and local health regions) where testing and treatment occur [28]. In BC, sexual health services are provided free of charge through regional or provincial health authorities (e.g., public health clinics and primary care programs), as well as by other clinics run by publicly-funded fee-for-service physicians and non-government organizations staffed by salaried nurses and physicians. STBBI testing and treatment options include screening (e.g., routine prenatal and/or gynecological care); testing, counseling, and partner notification (e.g., through physician-delivered primary care and public health programs); and more specialized sexual health services offered in particular settings (e.g., STBBI clinics; youth clinics; community health centres). Testing procedures vary depending on where they are accessed. Typically, face-to-face testing involves an initial consultation with a HCP to gather a client’s sexual history, conduct a physical exam, provide education and counseling, and collect specimens (blood, urine, swabs) [7]. Clinicians usually do sample collection on-site, but some may send clients to external specimen collection sites for blood and/or urine tests. Test results are normally available within seven to 10 days and the process of giving results varies across settings. HCP may tell clients to make a follow-up appointment to get their test results or only phone clients if they need to follow-up in person about their test results (i.e., a positive or inconclusive test result) and possible treatment [30]. It is into these multiple different contexts that GCO is being implemented.

### Theoretical framework

In this paper, we employ potentiality theory [31] from the field of anthropology to examine how the development of GCO was regarded by key agents involved in sexual health services (HCP, administrators, policymakers, and community-based sexual health service providers) to prospectively impact STI testing service provision for both providers and clients. Taussig and colleagues describe potentiality as “a hopeful idiom through which to imagine the benefits of new medical interventions”, while also often concurrently reflecting biomedicine’s “increasing anxiety about the negative potentials of life”, such as injury, disease, aging, or death at both the individual and population levels ([31], p. S4). Online health services are a rapidly growing component of public health provision, including testing for STBBI. In general, HCP, policymakers, and clients imbue online approaches with great positive potential (e.g., encouraging clients’ agency; providing testing to more clients in a cost-effective manner) [9]. However, the promise (i.e., the imagined benefits) of online health services may vary across contexts. Potentiality theory is especially useful for understanding context-based issues affecting implementation as it provides a useful framing to help unpack perceptions of GCO – be they positive, negative or ambiguous (e.g., enhanced appeal to young people; risks to ‘gold standard’ care provision; loss of provider control over an intervention; uncertainty regarding budget implications). Asking stakeholders about the anticipated effects of implementing this new intervention offers a way to examine potential gaps “between what is and what might, could, or even should be” ([31], p. S5). GCO’s hypothetical future can be articulated in positive, negative, or ambiguous ways, depending on how it is perceived. Understanding the ways in which the promise of GCO is perceived is a useful feature of context that provides another window into the ways in which context affects its continued implementation and adaptation.

Our aim in this paper is to examine how study participants’ attitudes and expectations about GCO reflect the context in which this intervention is being implemented, and provide insights into adaptations to the STBBI system that key stakeholders value. We also examine how interviewees anticipate GCO might affect the agency of STBBI testing clients. To the best of our knowledge, this is the first research to be published on the implementation context of an internet-based STBBI testing intervention. These data may provide important considerations not only for those involved in the implementation of GCO, but also others seeking to implement similar online STBBI testing services in other jurisdictions.

## Methods

### Data collection

Data for this paper are drawn from a four-year ethnographic study of the development and early implementation stages of GCO. We completed semi-structured, one-to-one interviews with 37 key agents (HCP, administrators, policymakers, and community-based sexual health service providers) involved in GCO’s development. Another study explored the opinions and expectations MSM had of GCO during its development [14], and an analysis of users’ experiences with the pilot test of GCO in Vancouver indicated that 13% of clients who created GCO accounts were under age 25, suggesting that the program may need to be adapted or promoted in different ways to increase uptake amongst youth [32]. Interviews were conducted during the pre-implementation phase of GCO in Vancouver in 2013, prior to its launch in September 2014. Participants were asked about their knowledge, perceptions, and experiences with online sexual health services. Their perceptions of GCO and its potential to function as a complementary service to existing face-to-face STBBI testing were examined, as was GCO’s perceived fit with existing clinical practices. A number of interview questions focused on aspects of the implementation context of GCO (e.g., which client populations might benefit the most or least from GCO; perceived complementarity of GCO with current practices at STBBI clinics and labs; cost considerations for changes to the STBBI system). (See Table 1 for Interview Guide questions.) We also conducted observations at 71 OSHS planning and implementation meetings to better understand GCO’s implementation context. Participants were recruited using purposive sampling and through participant observation activities. We selected participants who were directly involved in GCO’s development or implementation, as well as health and community service providers who do sexual health promotion.

**Table 1:** Interview Guide

Topics	Questions
Knowledge of online sexual health services	Please tell me what you know about: a) the provision of online health care within and outside of Canada. b) online sexual health services within and outside of the country. c) the BCCDC’s Online Sexual Health Services.
Perceived factors affecting the implementation of GCO	How feasible do you think the new GetCheckedOnline service will be with existing health human resource capacity in your health jurisdiction, clinic, or lab?
	What client populations do you think will benefit the most from the introduction of GCO? The least? Why?
	Would you recommend GCO to people who access your organization’s services? Why/why not?
	Tell me about any funding implications you think there might be for the new GetCheckedOnline service.
Perceived complementarity of GCO with current practices at STI/HIV testing clinics or labs	How well do you think the new GetCheckedOnline service might fit within your existing roles and responsibilities in the health jurisdiction, clinic, or lab where you work?
	How do you think GCO might affect the quality of care clinicians and lab technicians provide to clients seeking STI/HIV testing and treatment?
Perceived fit with extra-mural connections	How might GCO dovetail with (or clash with) clinical practice guidelines or accreditation requirements for clinics and labs in your health jurisdiction?
	Do you anticipate that GCO may identify a need to adapt or develop additional clinical practice guidelines or institutional regulations for STI/HIV testing services?
New training opportunities and other change management processes	What training opportunities do you think are needed in order to implement GCO in your health jurisdiction?
	How do you think this change to service provision can best be managed in your jurisdiction for you, your staff/co-workers, and clients?

Each participant was asked to provide written informed consent prior to being interviewed or included in participant observations. All study participants were asked to select a pseudonym (used to identify participants’ quotations in this paper). Thirty-four participants were interviewed in private rooms at their workplaces; the three remaining interviews were conducted by telephone due to distance or scheduling challenges. Each participant answered a five-item socio-demographic questionnaire before the semi-structured interview began. Interviews ranged in length from 40 min to over 2 hours, with the average duration being 75 min. The lead author, who has extensive training and experience conducting qualitative health research, conducted each interview (and in a few instances was assisted by another research staff member). Interviews were audio-recorded and transcribed verbatim. Participants had the opportunity to review their transcripts for any errors or revisions. Fieldnotes were prepared describing meeting observations, interview dynamics, and preliminary analyses [33]. The University of British Columbia’s Behavioural Research Ethics Board approved this study (certificate # H11–00547).

### Data analysis

Thematic analysis techniques from grounded theory [34] were employed to identify codes and broader discursive themes across the data. Research staff met regularly with the lead investigator to develop coding consensus and discuss newly emergent themes. The qualitative analysis software NVivo 10 was used to manage the data coding process [35]. Initial codes to organize the data into discrete categories were developed deductively through a combination of general themes informed by the literature and inductively through an in vivo reading of the data [33]. Sample initial codes included: human resource policies; privacy policies and legislation; and intramural and extramural influences on GCO’s development (all grouped under a broader theme of implementation context). We also developed codes to capture participants’ perceptions regarding the potentiality and promise of GCO (e.g., receptivity to GCO, feedback on the development of GCO, and anticipated effects on service provision post-implementation). As coding of the transcripts and fieldnotes progressed, we referred back to relevant policy and program documents we had analyzed at an earlier stage of our study. These documents provided additional macro-level context to the interview and observation data (e.g., participants’ references and interpretations of policies and best practice guidelines) and informed the later stages of our analysis and writing. During the final stage of coding (which is referred to as theoretical coding in grounded theory), we identified potentiality as the theoretical construct that explained study participants’ varied perceptions regarding their expectations about GCO [34]. As the drafting of this manuscript progressed, we frequently discussed our interpretations of the data to ensure consensus was reached.

## Results

The 37 interview participants ranged in age from 25 to 64 years. Fourteen participants were employed as HCP (nurses or physicians) who provide STBBI testing and treatment, while the remainder worked in healthcare administration (*n* = 7), policymaking (*n* = 4), community-based sexual health services (e.g., education, advocacy) (*n* = 6), or on the development of the BCCDC's OSHS program (n = 6).

As we began the initial phase of analysis, our reading of the discursive references in the data regarding the potential for GCO to improve STBBI service provision focused mostly on explicit statements about GCO’s “potential”; discussions about GCO’s possible impact on client agency and changes to service provision; and other contextual-based references (e.g., possible funding implications; impacts on “gold standard” service provision) to the intervention’s possible positive, negative, or ambiguous effects [31]. As the analysis continued, we were able to identify two broad thematic categories which summarize interviewees’ perspectives on GCO’s potential. First, improving access to STBBI testing; and second, impacts on STBBI service delivery post-implementation.

## Improving access to STBBI testing

In all of the interviews, participants said they believe GCO has the potential to improve the accessibility of STBBI testing in a number of ways, including (a) reducing waiting times; (b) providing enhanced privacy and confidentiality; and (c) directly appealing to more tech-savvy population subgroups. People who do not test because of long waiting times at clinics and young men who are uncomfortable seeking clinical care were regarded as ideal potential users of this low-barrier service. One public health administrator said:

It’s gonna stop people from having to sit in a waiting room with other people potentially that they don’t want to be around. … Even if it’s people they don’t know, people don’t like people to know about their sexual infections or that kind of thing. (‘Aidan’, administrator)

Most participants suggested that GCO’s developers prioritized client confidentiality and were following rigorous policy standards to ensure clients’ data were kept secure, which they perceived to be very important to most potential GCO users. A few administrators and clinicians expressed concern that a security breach *could* possibly jeopardize GCO users’ privacy, but countered that potentiality by noting the system’s features were built with privacy as an implementation and functional priority, and that GCO complied with the provincial government’s privacy legislation and operational framework. One salaried physician who specializes in sexual health service provision stated:

[T]hey’re being very cautious to protect the clients from [a privacy breach]. ‘Cause I think they just get [client code] numbers and such, like. So, I think, you know, I think the way they’re presenting it is done very well. Less privacy issues than just going to the clinic. (‘Elizabeth’, physician)

An emphasis on client confidentiality and privacy was regarded by some study participants as a factor that could encourage online testing as a ‘safer’ mode of testing (i.e., less risk of a confidentiality breach) than in-person testing.

Most participants said GCO would appeal to two priority groups: MSM and young people. GCO and the tech-savvy, proactive reputation of these groups were perceived as synergistic in terms of the intervention’s potential to enhance testing services. One administrator said:

This is going to be fantastic for MSM and youth! There’s not any question in my mind, because these are wider populations who are very comfortable being proactive and informed in their own care. (‘Marilyn’, administrator)

This comment reflects a common presumption that all young people and MSM are technologically savvy, have easy internet access, and are self-motivated to seek testing. This view was tempered slightly by some interview participants, who acknowledged that low-income individuals may not have affordable and reliable internet access, and some youth may not have private internet access. Most interviewees suggested that BC’s STBBI testing system will continue to need a diversity of approaches (GCO, clinics, outreach) in order to meet the overall population’s testing needs.

## Impacts on STBBI service provision post-implementation

Participants had conflicting perspectives on the value of GCO as it pertains to HCP productivity, conserving finite human resources, and budgetary constraints. Some participants stated that GCO may improve the productivity of the public STBBI system by shifting some clients to internet-based testing and away from more human resource-intensive face-to-face testing (including the services that they provide). These participants emphasized that they and the larger public health system can adapt to changing public demands when necessary, including during periods of fiscal constraint. In response to a question about the prospect of a funding shortfall should demand for STBBI testing increase as a result of the introduction of GCO, one administrator said:

I think it’s great that we hear [public health] labs freaking out about these costs because you know what? This is the true cost of providing the care that we should have been providing all along. Yeah, it’s a problem, but the people who need to figure it out will figure it out. Our job is to provide care … according to the clinical guidelines. Are we being successful by rationing care and being afraid of success and motoring on with these same terrible uncontrolled epidemics? *That’s* the failure. (‘Phinn’, administrator)

Many study participants suggested GCO would be a cost-effective alternative for people who test routinely and do not need to see a clinician every time. Some participants also suggested that GCO could reduce in-clinic testing demand by the so-called ‘worried well’ – people who are anxious about contracting an illness, despite often being perceived to be at low risk. These participants suggested GCO could provide the worried well with the reassurance they seek in a more cost-effective manner, rather than ‘wasting’ clinicians’ valuable time with face-to-face testing. One administrator said:

It allows people to get into testing but it also allows many of the worried well … the chance to get that reassurance that they want without necessarily impacting on a very limited resource in the system. (‘Marilyn’, administrator)

These participants predicted that by encouraging routine testers and the ‘worried well’ to use GCO, it would reducing wait times for people seeking in-person testing at STBBI clinics.

GCO developers also hoped that this intervention would appeal to MSM who test routinely. However, prior to its implementation, some HCP expressed concerns about this goal because GCO did not provide throat and rectal swab tests for chlamydia and gonorrhea. One clinician manager stated:

I think that there’s lots of people out there that we see, particularly in our gay male population, who don’t want to particularly talk to a nurse. They know the routine. … So they don’t really want to interface with us, right? They do it because they have to. …

The only thing I worry about around GetCheckedOnline is that there are no swabs, there are no throat or rectal swabs, and so I know it says that all over the [web]site … but people will assume they have been really checked when they haven’t been checked, right? (‘Arthur’, clinician manager)

GCO developers always planned to eventually introduce swab tests but this was not immediately feasible during the pilot phase because the laboratory collection centres were not structured to support client self-collection of swabs.

While most study participants acknowledged GCO’s potential to redirect some clients from in-clinic to online testing, some predicted that GCO might not decrease the demand for in-person testing services. One nurse said:

I think that it’s another tool that we can use to encourage people into normalized testing. I don’t see it as replacing a clinic visit, but enhancing or maybe introducing people to testing and maybe one day they feel comfortable coming in and seeing somebody for a full exam or a more thorough exam. (‘Chloe’, nurse)

Some participants also asserted that GCO would not be able to match clinicians’ expert knowledge. These participants believed that face-to-face STBBI testing is the ‘gold standard’ and suggested it may be the preferred form of testing for some clients. One administrator stated:

[GCO is] meant to supplement and guide people into the system [and] make it more efficient. … And people also go where they know the quality is, so if it turns out that they’re not getting quality services online and they think they’ll get their needs met better by going to the clinic, they’ll go to the clinic. (‘Phillip’, administrator)

Some clinicians expressed concern that demand for GCO might lead to a corresponding decrease in demand for in-clinic testing, resulting in job cuts at sexual health clinics or reduced fee-for-service visits at physicians’ general practice offices. However, most clinicians emphasized that online testing was not introduced into the system as a means to serve *all* populations or *replace* face-to-face testing. Rather, it was intended to complement existing clinic-based services, which were perceived to be already heavily utilized, as one OSHS developer stated:

[GCO] has pros and cons compared to clinical practice but it fills a niche that needs to be filled, right? Some people need to test more frequently and it’s really around convenience or accessibility: people who can’t get into a clinic, or people who *won’t* come into a clinic and [GCO] is how they’ll get tested. So I definitely see that it’s sort of filling a void and being really complementary to clinical services. (‘Mike’, OSHS developer)

Many interviewees also described online STBBI testing as an inevitable next frontier in the ever-expanding context of service provision. As one administrator commented, GCO reflects a broader trend towards patient-centred healthcare:

Either *we* make it available or people will just order it themselves. Like, this idea that we can control people the way we used to is ridiculous. I mean, they’ll order chlamydia kits from the UK. They’ll order gonorrhea kits from Mexico. It’s just gonna happen. So, my perspective is okay, let’s ensure some quality, opportunities for engagement, let’s monitor progress. … And that’s the perspective we need to take. (‘Marilyn’, administrator)

‘Marilyn’ supported GCO, in part, because she believed it would provide better quality of care than direct-to-consumer STBBI tests from outside of Canada. Moreover, she and a number of other study participants described GCO as having the potential to enhance the agency of people seeking testing by providing it online. However, GCO’s potential as a patient-centred form of healthcare created some uncertainty for some HCP who are accustomed to provider-controlled interventions. As previously noted, these clinicians regard provider-controlled interventions as providing the ‘gold standard’ of care. These clinicians asserted that the more detailed and nuanced nature of face-to-face, pre- and post-test counseling offers the highest standard of STBBI-related care and provides the added value of opening up discussions about other health issues, including referrals to other relevant services. Nonetheless, even though GCO was often not considered comparable to face-to-face testing, some regarded it as having the potential to empower users. As one GCO planner said:

[GCO] is a very self-directed approach, right? ... And I think there’s a sensibility out there, especially with young folks and what the internet can empower you to do. And you see this in the medical field. People think that they’re doctors because they, you know, googled something.... And so there’s that sometimes false belief that they know more than the doctor. But I think the good part of that, with [GCO], is that it puts their healthcare in their control and the feeling that comes with that is very powerful. You know, ‘I need to manage *myself* and I need to be in control of my own health. This is a tool that allows me to do that.’ I think that’s the crux of it. … It represents empowerment, I think, for health. (‘Rufus’, OSHS developer)

This perception that GCO has the potential to empower clients to take more proactive care of their sexual health gives the intervention significant symbolic value, which could be employed by its champions during its planned expansion to increase acceptance by HCP and uptake by users.

## Discussion

GCO is an intervention embedded in the social and structural processes of the public health system [36], and it is shaped by a varied range of attitudes and policies regarding healthcare provision standards. While many participants perceived this intervention would increase clients’ agency, it was also viewed with caution, particularly by a few HCP who were concerned by the prospect of a patient-centred (but not provider-controlled) intervention. These participants described GCO as a way for new clients to be introduced to STBBI testing and suggested that many users may ultimately seek out face-to-face testing, which they regarded as the gold standard of care. In contrast, GCO developers, policymakers, and administrators were more inclined to describe this intervention as a complement to existing in-person testing. This latter group emphasized GCO’s potential to increase patients’ agency and to empower BC’s STBBI testing system to expand its capacity.

The various agents in the public health system who participated in this study perceived that the implementation context into which GCO is being introduced is not static and they recognized that this intervention is a new way of ‘doing business’. This breakaway from the status quo created some uncertainty and can be regarded as embodying a set of potentialities which conflict with the standard conventions of provider-controlled STBBI testing and may eventually require adaptation by various agents in the system (e.g. shifting power dynamics at the service provision level; possible redistribution of testing resources). Other features of GCO were strongly aligned with a collective vision of ‘progress’, including public health policy’s shift to patient-centred care, increased patient access to services, and, ultimately, improved health outcomes. Some study participants noted that the STBBI testing system – as well as the broader health care system – is capable of adapting to these possible changes. Many study participants noted that GCO may also need to adapt as it expands to other regions of the province and seeks to meet the needs of local jurisdictions (e.g., adaptations to treatment provision). Hence, the symbolic value of the potential and promise of GCO could emerge as a powerful influence on its acceptance and eventual uptake by both healthcare providers and users across various contexts, regardless of the (real) multiple and multiplied effects, which could vary from place to place.

Study participants agreed that the status quo for STBBI testing and treatment in BC is not ideal. STI incidence rates are rising and people often do not receive testing and treatment in a timely manner. Participants wanted GCO to succeed, even if they were uncertain of the extent to which it might trigger adaptations to testing and treatment provision. The potential changes GCO might generate “creates a world on which realities travel in spite of unknown feasibility” ([37], p. S34), eliciting a range of reactions from participants. GCO’s developers are well aware that some might be fearful of the changes this new intervention brings (e.g., some HCP prefer provider-controlled STBBI service provision), while others might expect more than can be delivered, particularly early in the program. From the outset, GCO has been promoted as ‘another tool in the toolbox’, which also served as a way to manage expectations while concomitantly demonstrating GCO’s potential feasibility and success within the larger sexual healthcare system. For example, GCO initially lacked the capacity to provide throat and rectal swab tests for gonorrhea and chlamydia – a feature HCP regarded as critical for MSM clients. It was feasible for GCO to incorporate throat and rectal swab tests based on this feedback from clinicians – and these tests were added to the overall ‘toolbox’ available in either online or clinic settings [27].

GCO is the first comprehensive internet-based service in Canada to offer testing for STBBIs, and one of a limited number in the world. To date, most research related to internet-based testing services has been focused on demonstrating acceptability, uptake, and testing outcomes among populations targeted during the immediate pre- and post-implementation period, without broader consideration of impacts on providers and health systems [38–42]. Qualitative research to understand how these services are perceived and their potential to overcome testing barriers has largely been hypothetical (i.e., before a program is in development) and focused on potential end-users or clients of the service. To our knowledge, only one other published study has considered the views of healthcare workers regarding internet-based testing [43]. In this UK study examining internet-based chlamydia screening for heterosexual men in general, interviews with physicians and public health nurses demonstrated broad support for a planned nationwide program. Similar to our study, HCPs in the UK-based study regarded the planned screening program as having the potential to appeal to young men because it would be convenient, easy to access, and more private and anonymous than clinic-based testing [43]. In our study, we asked key stakeholders to go beyond describing GCO’s potential to appeal; we also engaged with them in discussions about possible adaptations of GCO post-implementation, including the development of strategies to adapt GCO within the existing STBBI system (e.g., with minimal disruption or added burden to clinic staff work loads) as well as the identification of key adaptations to GCO (e.g., introducing swab tests to GCO) that synergize with the existing system. Participants also identified how the larger system itself could react (e.g., increases in costs and wait times as testing extends to more people). They also identified what would be considered a ‘game-changer’ to the system. GCO not only empowers consumers; providers have relinquished their previous level of control. This change to the drivers of the system requires a new “perspective” (in ‘Marilyn’s’ words). It’s a change to the system’s paradigm and mindset, and hence it is widely considered to be transformative [44].

### Recommendations for future research

Conventional health intervention research has focused primarily on examining the effects of an intervention, often overlooking the context in which interventions occur [36, 45, 46]. This is particularly true for internet-based health interventions, where research has focused primarily on acceptance of technology and proof-of-concept or feasibility studies, with few evaluations taking into consideration the agentic practices of various agents (e.g., client empowerment; HCP-controlled service provision) within implementation contexts [10, 47–50]. We demonstrate that HCP, policymakers, and community stakeholders’ attitudes and expectations about GCO prior to its implementation can provide valuable insights into how it should be implemented, influencing the potentiality and promise of this service. We recommend that evaluators of internet-based health interventions deliberately seek to understand contextual factors influencing implementation, as one component of an overall program of research to assess the effectiveness of these interventions. We have adopted this approach for GCO, where research and evaluation has been embedded at all phases of development and implementation [27].

Since the pre-implementation interviews were conducted, the roll-out of GCO has progressed in a deliberate and controlled manner, with OSHS developers making changes to the intervention when challenges have arisen or from research findings [27, 32]. GCO is now in the implementation and scale-up phase, following demonstration of proof-of-concept and feasibility, leading to adoption by two other health regions in BC in 2016. This points to the need for longer-term research, monitoring, and follow-up of GCO program aims (e.g., rates of testing), as well as key features of the implementation context (e.g., geographic considerations; jurisdiction issues; cost-effectiveness in reaching new groups, total cost of the innovation, and funding implications for the system; community-based sexual health service provision) and local user experiences in order to inform subsequent adaptations. Future research examining what activities GCO may displace in the public health practice system and any subsequent effects would also be beneficial [51], as it could help inform improvement of GCO and its expansion into other settings. Our results also illustrate the importance of considering clients’ agency when developing this intervention and have informed our current study examining the experiences of GCO users. Early results of this latter research have been published [32], and further research will be published in the near future.

### Strengths and limitations

Findings from this qualitative study provide insights into some of the anticipated benefits and challenges of implementing a publicly funded internet-based STBBI testing service in BC. In addition to university-based researchers, our research team included physician and operations leads involved with GCO at the BCCDC who were known to many of the individuals interviewed in this study. Only the university-based team members conducted data collection and analysis and had access to the full transcripts. To minimize social desirability bias during the informed consent process, participants employed at the BCCDC were assured that their decision of whether or not to participate in the study would in no way affect their employment, and that BCCDC research team members would not be informed of their real identities. In addition, BCCDC-based members of the research team engaged HCP, policy makers, and community stakeholders in all stages of the development of GCO (including inviting them to participate in this study). Ensuring the ongoing involvement of stakeholders was integral to the design and early implementation of this intervention and contributed to the feasibility and efficacy of GCO.

Our study also has some limitations. Interviews were completed with HCP, administrators, and community stakeholders primarily in Vancouver, and they were conducted prior to the implementation of GCO. Interviews were conducted with HCP who were familiar with, or involved in, the development of GCO model; therefore, our study may not reflect the perceptions of providers who were unfamiliar with this service [52] and may be biased toward a positive view of the intervention’s anticipated benefits. Our research team initially planned to conduct follow-up interviews with key agents post-implementation. However, lengthy delays in the development and implementation of GCO (e.g., restructuring of the provincially-funded technical support service that provides service to the BCCDC) beyond the funding period for this study made follow-up interviews unfeasible. Given that our study examined how the development of GCO was regarded by key agents involved in sexual health service provision in Vancouver, British Columbia, the relevance of our study findings to other internet-based testing programs or health interventions may be limited.

## Conclusion

The majority of participants emphasized the anticipated benefits of GCO over its potential challenges and risks. Key stakeholders believed GCO has the potential to reduce waiting times, to enhance privacy and confidentiality for clients hesitant to access in-person testing, to appeal to tech-savvy population subgroups, and to optimize and/or redistribute finite resources to provide testing to more people, more often. Overwhelmingly, study participants perceived that the introduction of GCO might trigger adaptations within its implementation contexts, such as the redistribution of testing resources and changing power dynamics at the service provision interface. Study participants also noted that implementation contexts are not in a static state; changes within other parts of the STBBI testing system, or indeed the broader health care system (e.g., re-allocation or reduction of resources), may trigger the need for adaptation within GCO. This dialectical view of adaptation used by GCO’s developers as they continue to engage various agents is key to realizing the potential and promise of GCO across BC and to determining relevance for other jurisdictions.

## Abbreviations

BC: British Columbia
BCCDC: British Columbia Centre for Disease Control
GCO: GetCheckedOnline
HCP: Healthcare provider
HIV: Human immunodeficiency virus
MSM: Men who have sex with men
OSHS: Online sexual health services
STBBI: Sexually transmitted and other blood-borne infections
STI: Sexually transmitted infection(s)
